# Variations in the bitterness perception-related genes *TAS2R38* and *CA6* modify the risk for colorectal cancer in Koreans

**DOI:** 10.18632/oncotarget.15512

**Published:** 2017-02-19

**Authors:** Jeong-Hwa Choi, Jeonghee Lee, Jae Hwan Oh, Hee Jin Chang, Dae Kyung Sohn, Aesun Shin, Jeongseon Kim

**Affiliations:** ^1^ Molecular Epidemiology Branch, Division of Cancer Epidemiology and Prevention, National Cancer Center, Ilsandong-gu, Goyang-si, Gyeonggi-do, 10408, Korea; ^2^ Center for Colorectal Cancer, National Cancer Center Hospital, National Cancer Center, Ilsandong-gu, Goyang-si, Gyeonggi-do, 10408, Korea; ^3^ Department of Preventive Medicine, Seoul National University College of Medicine, Jongno-gu, Seoul, 03080, Korea

**Keywords:** bitterness perception, CA6, colorectal cancer, dietary intake, TAS2R38

## Abstract

Bitterness perception is known to be an important factor in individuals’ dietary behaviors and is also associated with the sensing of nutritious/noxious molecules for subsequent metabolic responses in multiple organs. Therefore, the genetic variation in bitterness sensing may be associated with diet-related diseases, including colorectal cancer (CRC). We investigated the influence of variations in the bitterness-sensing genes taste receptor type 2 member 38 (*TAS2R38*) and carbonic anhydrase 6 (*CA6*) on the consumption of food, tobacco and alcohol and the risk of CRC in Koreans. The study population consisted of 681 cases and 1361 controls, and their intake of vegetables, fruits, fiber, fat-food and sweets was analyzed. The genotypes for *TAS2R38* A49P, V262A and I296V and CA6 rs2274333 A/G were assessed using the MassArray technique. Our findings suggested that the *TAS2R38* diplotype, *CA6* rs2274333 and their combined genotype had a negligible influence on dietary and alcohol intake. The combined *TAS2R38-CA6* AVI/AVI-AA genotype was associated with higher tobacco consumption than the other genotypes in CRC cases only. However, the genetic variations were a significant risk factor for CRC. The *TAS2R38* AVI/AVI diplotype and *CA6* G allele were associated with a reduced risk of CRC. Moreover, when the combined genotypes of the subjects were analyzed, possessing both the variant diplotype/variant allele (AVI/AVI+G*) was associated with a greater reduction in the risk of CRC (adjusted OR = 0.49; 95%CI: 0.34–0.74). In summary, variations in the bitterness perception genes *TAS2R38* and *CA6* did not influence the examined food intake in Koreans. However, those genetic variants were a decisive modifying factor of CRC susceptibility.

## INTRODUCTION

Colorectal cancer (CRC) has been a major health concern in the Western hemisphere and is currently an emerging issue in Korea. Although the recent mortality rate of CRC seems to have stabilized or declined, the age-adjusted incidences of CRC for men and women were 45.6 and 24.4 per 100,000 in 2013, respectively, making CRC the third most common type of cancer in Korea [1]. Evidence suggests that in addition to common environmental factors, including nutrition and dietary intake, an individual's genetic background is a critical component of CRC etiology [2]. Therefore, genetic variants in taste perception, especially bitterness sensing and its influence on dietary intake, are considered significant risk factors for CRC susceptibility. Because bitterness is a key determinant in the rejection/acceptance of food products, genetically modulated sensitivity to bitterness intensity may lead to an individual's differential intake of dietary and consumer goods, which may further be linked to the risk of diet-related diseases [3].

The perception of the bitter thiocyanate (N-C=S) moiety as tested with 6-n-propylthiouracil (PROP) and phenylthiocarbamide (PTC) has been studied extensively as a human tasting trait with respect to dietary behavior and health outcomes [3]. The variability of PROP/PTC bitterness sensation among individuals is known to be associated with the taste receptor type 2 member 38 (*TAS2R38*, T2R38) gene, and its haplotype consists of three common missense variations (A49P, V262A and I296V). Individuals with the PAV haplotype are sensitive to the bitterness of PROP/PTC (taster), whereas those who possess the AVI/AVI haplotype are not (non-taster) [4]. The *TAS2R38* diplotype was therefore observed to be associated with an individual's differential intake of bitter tasting beverages, cruciferous vegetables (which are high in glucosinolates containing thiourea moieties), fruits and blood folate concentrations [3, 5–8]. Furthermore, the *TAS2R38* diplotype has been independently associated with the risk of gastrointestinal cancer regardless of modifications in dietary food intake [9–11]. The sensing of harmful molecules by the gustatory T2R38 may initiate subsequent protective responses, such as the neutralization or expulsion of carcinogenic molecules from the alimentary track. Therefore, the differential T2R38 activity may modify the risk of CRC independent of dietary intake [12]. This diet-independent disease risk-modifying effect of T2R is also supported by evidence from extra-gastrointestinal tract tissues. In cells in the upper respiratory system, T2R38 responds to quorum-sensing molecules secreted by gram-negative bacteria and is associated with the activation of nitric oxide production and airway infection [13]. T2R receptors are also expressed on the thyroid, which is the center of endocrine metabolism, and regulate thyrocyte function and triiodothyronine and thyroxine production [14]. These findings suggest that *TAS2R38* genetic variants may be a genetic marker for organ or metabolic function as well as bitterness sensing [10, 11]. However, some controversy still exists: the *TAS2R38* haplotype does not completely describe the differential intensity of bitterness and dietary intake among individuals, and the association between the *TAS2R38* diplotype, dietary consumption and CRC risk also differs among ethnicities [11, 15]. These findings suggest that other genetic components may need to be considered to better understand the mechanism of bitterness perception and the related susceptibility to CRC.

Carbonic anhydrase VI (CAVI, gustin, *CA6*), a zinc-containing salivary protein, is known to be involved in PROP-mediated bitterness sensation [16–18]. CA family proteins play a central role in pH regulation and electrolyte balance, and CAVI is known to be a trophic factor for the growth and development of taste buds and bitterness sensation [19, 20]. One sequence variation in CAVI (rs2274333, A/G, S90G) was recently reported to modify protein function. Individuals with a variant G allele showed differential fungiform papillae density and morphology, and the variant G allele modified the intensity of bitterness both on its own and in concert with the *TAS2R38* haplotype [16]. The variant CAVI protein also showed an association with the risk of caries and other oral health parameters [21, 22]. Moreover, some CA isozymes are thought to be associated with unregulated cell proliferation and malignancy invasion [23, 24]. Considering these regulatory roles of CAVI in cellular homeostasis and bitterness sensation, variant CAVI may be a critical modifying factor for dietary intake and gastrointestinal dysfunctions [25]. However, the epidemiological evidence for the modifying effect of variant CAVI in dietary intake and its pathological role in gastrointestinal diseases has not been fully investigated.

This study examined whether the bitter taste receptor proteins T2R38 and CAVI influence the dietary, alcohol and tobacco consumption of Koreans. The role of genetic variations in *TAS2R38* and *CA6* in the development of CRC was also investigated.

## RESULTS

### General characteristics of the study population

The general characteristics of the study subjects showed significant differences depending on CRC phenotype (Table 1). CRC was more likely to develop in former drinkers and in those with a lower body mass index and lower education and income levels. Patients with CRC were also less likely to regularly exercise and were more likely to live alone and to have a family history of CRC. These differences are known to modify the risk of CRC; therefore, such variables were included in the subsequent analyses as potential confounders. Lastly, the dietary zinc intake was determined because CAVI is a zinc-dependent metalloprotein. Since the controls and CRC patients exhibited significant differences in dietary zinc, the dietary zinc intake was adjusted for in the statistical models of *CA6* genetic variation. However, there was no significant difference in the dietary zinc intake among the *CA6* genotypes (data not shown).

**Table 1:** Descriptive data of the study population by colorectal cancer phenotype

	Total	Cases	Controls	*p^a^*
Number of participants (%)^b^	2,042 (100)	681 (33.4)	1361 (66.7)	
Sex (%)				0.955
Male	1,390 (68.1)	463 (68.0)	927 (68.1)	
Female	652 (31.9)	218 (32.0)	434 (31.9)	
Age (mean, year)	56.1 ± 9.3	56.5 ± 9.6	56.0 ± 9.1	0.258
Body mass index (kg/m^2^)				0.437
< 25	1,374 (67.3)	466 (68.4)	908 (66.7)	
≥ 25	668 (32.7)	215 (31.6)	453 (33.3)	
Tobacco smoking (%)				0.088
Never	909 (44.5)	308 (45.2)	601 (44.2)	
Former	742 (36.3)	228 (33.5)	514 (37.8)	
Current	391 (19.15)	145 (21.3)	246 (18.1)	
Alcohol drinking (%)				0.001
Never	619 (30.3)	208 (30.5)	411 (30.2)	
Former	220 (10.8)	97 (14.2)	123 (9.0)	
Current	1,203 (58.9)	376 (55.2)	827 (60.76)	
Regular exercise (%)				< .001
Yes	1,029 (50.4)	217 (31.9)	812 (59.6)	
No	1,006 (49.3)	646 (68.1)	542 (39.8)	
Missing	7 (0.3)	-	7 (0.5)	
First-degree family history of colorectal cancer (%)				0.039
Yes	127 (6.2)	53 (7.8)	74 (5.4)	
No	1,915 (93.8)	628 (92.2)	1,287 (94.6)	
Marital status (%)				< .001
Married or with a partner	1,800 (88.2)	576 (84.6)	1,224 (89.9)	
Single	171 (8.4)	82 (12.0)	89 (6.5)	
Never married	65 (3.2)	21 (3.1)	44 (3.2)	
Missing	6 (0.3)	2 (0.3)	4 (0.3)	
Education level (%)				< .001
Middle school graduate or less	439 (21.5)	249 (36.6)	190 (14.0)	
High school graduate	855 (41.9)	261 (38.3)	594 (43.6)	
College graduate or more	739 (36.2)	171 (25.1)	568 (41.7)	
Missing	9 (0.4)	-	9 (0.7)	
Household income (10,000 won/month)				< .001
< 100	176 (8.6)	91 (13.4)	85 (6.3)	
100–200	373 (18.3)	143 (21.0)	230 (16.9)	
201–400	883 (43.2)	301 (44.2)	582 (42.8)	
> 400	570 (28.0)	146 (21.4)	424 (31.2)	
Missing	40 (2.0)	-	40 (2.9)	
Dietary zinc intake (mg/day)	8.4 ± 1.6	8.1 ± 1.4	8.5 ± 1.6	< .001

^a^*P*-values present the difference between cases and controls. Age and zinc intake were examined using Student's *t*-tests; other variables were assessed using chi-square analyses.

^b^Numbers in parentheses are percentages; other data are presented as the means ± standard deviation.

### Distribution of *TAS2R38* and *CA6* genetic variations and the combined genotype

Table 2 presents the distribution of the *TAS2R38* diplotype, the *CA6* rs2272333 genotype and their combined genotype. All genetic variants were in Hardy-Weinberg equilibrium (*P* > 0.05). The three genetic variants in *TAS2R38* (A49P, V262A and I296V) were highly correlated each other (*r*^2^ > 0.99). A total of 6 haplotypes were present in the current Korean population. The most frequently observed haplotypes were PAV and AVI, and their combinations (PAV/PAV, PAV/AVI and AVI/AVI) described the majority of the *TAS2R38* diplotypes (99.7%). Four other haplotypes (AAV, AVV, PVI and PVV) and diplotypes (AAV/AVI, PAV/AVV, PVI/AVI and PVV/AVI) were also computed for the current population. However, due to the rarity of these diplotypes (0.3%, *n* = 6), these subjects were excluded from the subsequent statistical investigation. The chi-squared test showed that the distributions of the three diplotypes (PAV/PAV, PAV/AVI and AVI/AVI, co-dominant model) were not associated with CRC outcome. However, when the subjects were classified based on the presence of the PAV haplotype (PAV/* vs. the AVI/AVI, recessive model), the distribution of diplotypes differed by CRC phenotype (*p*_chisq_ = 0.022).

**Table 2:** Distribution of the *TAS2R38* diplotype, *CA6* rs2274333 genotype and the combined genotype

	Total (*n* = 2,042)^a^	Case (*n* = 681)	Control (*n* = 1,361)	*p*_chisq_^b^
*TAS2R38* diplotype				
PAV/PAV	726 (35.6)	251 (36.9)	475 (34.9)	0.074
PAV/AVI	981 (48.0)	337 (49.5)	644 (47.3)	
AVI/AVI	329 (16.1)	92 (13.5)	237 (17.4)	
AAV/AVI	3 (0.15)	-	3 (0.2)	
PAV/AVV	1 (0.05)	1 (0.2)	-	
PVI/AVI	1 (0.05)	-	1 (0.1)	
PVV/AVI	1 (0.05)	-	1 (0.1)	
PAV/PAV+PAV/AVI^c^	**1,707 (83.6)**	**588 (86.5)**	**1,119 (82.2)**	**0.022**
*CA6* rs2274333				0.009
AA	353 (17.3)	142 (20.9)	211 (15.5)	
GA	957 (46.9)	299 (43.9)	658 (48.4)	
GG	732 (35.9)	240 (35.2)	492 (36.2)	
GA + GG^d^	**1,689 (82.8)**	**539 (79.1)**	**1,150 (84.6)**	**0.003**
Combined genotype (*TAS2R38*+*CA6*)^e^				0.002
PAV/*+AA	288 (14.2)	119 (17.5)	169 (12.5)	
PAV/*+G*	1,419 (69.7)	469 (69.0)	950 (70.1)	
AVI/AVI+AA	65 (3.1)	23 (3.3)	42 (3.0)	
AVI/AVI+G*	264 (13.0)	69 (10.2)	195 (14.4)	

^a^‘*n*’ indicates numbers of subjects.

^b^*P*-values from chi-square tests.

^c^Comparison for the distribution of the recessive model (PAV/PAV+PAV/AVI versus AVI/AVI) by colorectal cancer phenotype.

^d^Comparison for the distribution of the dominant model (AA versus GA+GG) by colorectal cancer phenotype.

^e^Combined genotype of recessive models for the *TAS2R38* diplotype and dominant model for *CA6* rs2274333, PAV/*+G*=[*TAS2R38* (PAV/PAV+PAV/AVI)+*CA6* (GA+GG)], AVI/AVI+G*=[*TAS2R38* (AVI/AVI) + *CA6* (GA+GG)], PAV/*+AA=[*TAS2R38* (PAV/PAV+PAV/AVI)+*CA6* (AA)] and AVI/AVI+AA=[*TAS2R38* (AVI/AVI)+*CA6* (AA)].

The distribution of the *CA6* rs22274333 genotype is also shown in Table 2. The G allele was more frequent in the entire and control population than the A allele, with frequencies of 0.59 and 0.60, respectively. However, evidence has suggested that the substitution of adenine to guanine leads to the structural alteration of the CAVI protein, thereby reducing both the protein activity and bitterness intensity [26, 27]. Since the taster haplotype PAV is considered the dominant haplotype for *TAS2R38* and to better estimate the effect of reduced bitterness sensitivity on dietary intake and disease risk, we considered adenine (taster allele) as the reference allele for this study. A chi-squared evaluation revealed that the distribution of the *CA6* rs2274333 genotype differed between CRC cases and controls (*p*_chisq_ = 0.009). This genotype-CRC association was also retained when the subjects were grouped according to the presence of the variant G allele (*p*_chisq_ = 0.003, AA vs. G*).

The numbers of groups or subgroups with limited numbers of subjects could obscure the associations between genetic variants and phenotypic outcomes. Therefore, to establish the *TAS2R38-CA6* combined genotype, the results of chi-squared tests were applied. Subjects were classified based on the presence of taster PAV/* (recessive model) and AA (dominant model). Four combinations (PAV/*+AA, PAV/*+G*, AVI/AVI+AA, AVI/AVI+G*) were evident in the current study subjects, and the distributions of these combinations differed distinctively by CRC phenotype (*p*_chisq_ = 0.002). This combined genotype was therefore applied for subsequent statistical analyses (the distributions of all combined genotypes are presented in Supplementary Table 1).

### Genetic variations and dietary intake, alcohol and tobacco consumption

The effect of *TAS2R38* and *CA6* genetic variants and their combined genotype on the intake of total energy, all vegetables, cruciferous vegetables, dark green vegetables, all fruits, citrus fruits, fiber, fat-food, sweets, alcohol and tobacco were examined, and the results are presented in Tables 3, 4 and 5. Tables 3 and 4 present the mean consumption of the evaluated variables for each of the *TAS2R38* and *CA6* genetic variants in all subjects as well as in CRC cases and controls, respectively. Generalized linear models and Student's *t*-tests, however, revealed no significant differences in the variables among the genotypes either in the presence or absence of confounders in any of the examined groups of subjects. The only marginal association was observed in a recessive model between the *TAS2R38* diplotype and tobacco intake in CRC cases (*p* = 0.046).

**Table 3:** Mean consumption of selected foods, alcohol and tobacco for the TAS2R38 diplotype in all subjects and colorectal cancer cases and controls (mean ± standard deviation)^a^

TAS2R38	Energy (kcal/day)	Vegetables (g/day)			Fruits (g/day)		Fiber (g/day)	Fat-food (g/day)	Sweets (g/day)	Alcohol^b^ (g/day)	Tobacco^b^ (cigarettes/day)
		All	Cruciferous	Dark green	All	Citrus					
All subjects (*n* = 2036)											
PAV/PAV (*n* = 726)	1992.1 ± 640.9	373.1 ± 191.7	179.6 ± 113.5	38.3 ± 36.2	191.8 ± 193.7	40.8 ± 61.5	19.5 ± 6.8	4.8 ± 4.4	24.1 ± 31.2	22.9 ± 31.6	17.2 ± 9.5
PAV/AVI (*n* = 981)	1963.8 ± 636.4	372.3 ± 186.5	176.7 ± 104.8	38.0 ± 34.3	177.8 ± 178.2	38.3 ± 49.4	19.3 ± 6.3	4.7 ± 4.9	26.8 ± 44.4	23.1 ± 30.5	16.3 ± 9.0
AVI/AVI (*n* = 329)	1975.7 ± 618.3	383.8 ± 197.3	177.9 ± 117.2	39.3 ± 29.3	196.2 ± 226.2	40.8 ± 54.2	19.9 ± 6.6	4.5 ± 4.6	27.6 ± 42.3	18.6 ± 23.8	16.4 ± 9.5
*p*_crude_^c^	0.595	0.787	0.862	0.432	0.207	0.830	0.446	0.275	0.445	0.149	0.266
*p*_adjusted_^d^	0.303	0.974	0.928	0.634	0.281	0.928	0.739	0.422	0.580	0.280	0.293
*p*_ttest_^e^	0.901	0.557	0.659	0.191	0.568	0.700	0.223	0.237	0.649	0.053	0.364
Controls (*n* = 1,356)											
PAV/PAV (*n* = 475)	1841.0 ± 571.2	387.7 ± 207.8	186.6 ± 124.0	41.6 ± 39.3	206.1 ± 204.7	43.2 ± 64.9	20.3 ± 7.2	4.5 ± 4.3	23.2 ± 29.2	17.4 ± 22.4	16.2 ± 8.1
PAV/AVI (*n* = 644)	1833.5 ± 615.3	393.2 ± 206.6	182.5 ± 113.8	42.1 ± 39.0	191.4 ± 197.1	38.9 ± 53.8	20.3 ± 6.8	4.5 ± 5.1	26.1 ± 46.3	17.4 ± 21.7	15.3 ± 8.1
AVI/AVI (*n* = 237)	1867.0 ± 601.6	396.7 ± 212.0	180.6 ± 124.2	42.0 ± 32.0	216.1 ± 250.5	43.2 ± 58.4	20.6 ± 7.1	4.4 ± 4.3	27.2 ± 43.6	16.7 ± 21.6	14.8 ± 8.3
*p*_crude_	0.618	0.738	0.769	0.877	0.091	0.314	0.876	0.885	0.441	0.814	0.109
*p*_adjusted_	0.509	0.923	0.332	0.781	0.090	0.332	0.855	0.978	0.661	0.710	0.063
*p*_ttest_	0.462	0.933	0.497	0.606	0.204	0.240	0.624	0.844	0.475	0.541	0.072
Cases (*n* = 680)											
PAV/PAV (*n* = 251)	2278.1 ± 668.7	345.5 ± 153.5	166.2 ± 88.9	32.1 ± 28.6	164.9 ± 168.0	36.1 ± 54.2	17.9 ± 5.6	5.4 ± 4.6	25.8 ± 34.8	34.1 ± 42.6	19.3 ± 11.5
PAV/AVI (*n* = 337)	2212.8 ± 601.7	332.5 ± 131.7	165.8 ± 84.2	30.0 ± 20.6	152.3 ± 132.1	37.2 ± 39.6	17.5 ± 4.6	5.0 ± 4.5	28.1 ± 40.5	34.2 ± 40.5	18.2 ± 10.3
AVI/AVI (*n* = 92)	2255.7 ± 573.8	350.4 ± 149.0	170.7 ± 97.1	32.4 ± 19.3	145.1 ± 134.2	34.1 ± 39.9	18.1 ± 5.0	4.7 ± 5.3	28.7 ± 39.2	23.9 ± 28.7	20.9 ± 11.0
*p*_crude_	0.603	0.669	0.961	0.536	0.440	0.377	0.632	0.139	0.852	0.162	0.156
*p*_adjusted_	0.423	0.589	0.341	0.339	0.288	0.341	0.634	0.105	0.892	0.251	0.098
*p*_ttest_	0.647	0.534	0.873	0.248	0.240	0.258	0.411	0.128	0.913	0.070	0.046

^a^Subjects with the AAV/AVI (*n* = 3); PAV/AVV (*n* = 1), PVI/AVI (*n* = 1), and PVV/AVI (*n* = 1) diplotypes were excluded from the statistical analyses due to the limited numbers.

^b^The mean consumption was estimated among current/former drinkers and smokers.

^c^*P*-values for crude generalized linear models compared the intake of selected foods, alcohol and tobacco among groups.

^d^*P*-values for generalized linear models adjusted for sex, age, body mass index, smoking, alcohol drinking, regular exercise, family history of colorectal cancer, marital status, education level and household income as appropriate.

^e^*P*-values from Student’s *t*-tests of the comparison between genotypes of the recessive model for *TAS2R38* (PAV/PAV+PAV/AVI versus AVI/AVI).

**Table 4:** Mean consumption of selected foods, alcohol and tobacco for the CA6 rs2274333 genotype in all subjects and colorectal cancer cases and controls (mean ± standard deviation)

CA6 rs2274333	Energy (kcal/day)	Vegetables (g/day)			Fruits (g/day)		Fiber (g/day)	Fat-food (g/day)	Sweets (g/day)	Alcohol^a^ (g/day)	Tobacco^a^ (cigarettes/day)
		All	Cruciferous	Dark green	All	Citrus					
All subjects (*n* = 2042)											
AA (*n* = 353)	2031.8 ± 661.5	374.5 ± 198.8	175.6 ± 109.0	37.5 ± 36.8	190.4 ± 221.5	43.9 ± 62.7	19.2 ± 6.5	4.6 ± 5.0	23.7 ± 30.2	23.4 ± 29.3	17.6 ± 10.7
GA (*n* = 957)	1965.2 ± 638.2	376.9 ± 193.0	179.1 ± 114.4	39.5 ± 34.3	183.8 ± 190.2	37.6 ± 52.0	19.5 ± 6.5	4.7 ± 4.6	26.1 ± 38.6	21.9 ± 30.0	16.1 ± 8.7
GG (*n* = 732)	1957.4 ± 617.7	372.3 ± 183.8	177.8 ± 104.4	37.8 ± 34.7	186.3 ± 179.5	40.0 ± 54.1	19.5 ± 6.6	4.8 ± 4.6	26.8 ± 45.1	22.4 ± 30.2	16.9 ± 9.3
*p*_crude_^b^	0.199	0.917	0.820	0.226	0.399	0.453	0.673	0.687	0.789	0.722	0.174
*p*_adjusted_^c^	0.185	0.959	0.256	0.487	0.158	0.256	0.924	0.690	0.716	0.989	0.347
*p*_ttest_^d^	0.073	0.815	0.555	0.149	0.521	0.429	0.381	0.406	0.532	0.493	0.143
Controls (*n* = 1,361)											
AA (*n* = 211)	1828.9 ± 575.1	400.0 ± 222.5	183.9 ± 121.0	41.5 ± 41.3	210.6 ± 260.4	46.1 ± 71.6	20.3 ± 7.1	4.6 ± 5.1	22.6 ± 28.2	16.5 ± 21.2	15.1 ± 6.8
GA (*n* = 658)	1843.7 ± 614.3	393.5 ± 207.3	185.5 ± 122.8	43.5 ± 37.2	195.1 ± 199.5	38.6 ± 52.0	20.4 ± 6.8	4.4 ± 4.6	24.5 ± 35.2	16.8 ± 22.0	15.2 ± 8.2
GG (*n* = 492)	1838.7 ± 586.3	387.7 ± 204.5	181.2 ± 113.5	40.9 ± 39.8	204.3 ± 199.4	42.2 ± 60.7	20.4 ± 7.3	4.6 ± 4.5	27.4 ± 50.5	18.3 ± 22.1	16.1 ± 8.6
*p*_crude_	0.998	0.750	0.844	0.268	0.311	0.685	0.963	0.622	0.847	0.418	0.411
*p*_adjusted_	0.797	0.662	0.390	0.416	0.121	0.380	0.899	0.669	0.893	0.388	0.487
*p*_ttest_	0.953	0.607	0.879	0.440	0.674	0.893	0.783	0.911	0.624	0.645	0.811
Cases (*n* = 681)											
AA (*n* = 142)	2333.1 ± 668.4	336.6 ± 150.1	163.4 ± 87.3	31.6 ± 27.8	160.4 ± 141.4	40.3 ± 45.3	17.7 ± 5.2	4.7 ± 4.8	25.4 ± 32.9	33.6 ± 36.2	21.5 ± 14.2
GA (*n* = 299)	2232.6 ± 608.7	340.3 ± 151.0	164.9 ± 91.9	30.8 ± 24.9	159.1 ± 166.0	35.4 ± 52.1	17.8 ± 5.3	5.3 ± 4.5	29.5 ± 45.1	34.0 ± 41.0	18.0 ± 9.4
GG (*n* = 240)	2200.7 ± 610.4	340.9 ± 126.1	170.8 ± 82.4	31.2 ± 19.1	149.3 ± 121.8	35.3 ± 35.5	17.7 ± 4.5	5.0 ± 4.8	25.7 ± 31.2	31.2 ± 41.4	18.7 ± 10.5
*p*_crude_	0.183	0.754	0.333	0.488	0.977	0.481	0.977	0.212	0.661	0.404	0.265
*p*_adjusted_	0.061	0.810	0.377	0.617	0.904	0.377	0.910	0.328	0.800	0.555	0.166
*p*_ttest_	0.095	0.541	0.515	0.427	0.840	0.267	0.851	0.155	0.377	0.450	0.125

^a^The mean consumption was estimated among current/former drinkers and smokers.

^b^*P*-values for crude generalized linear models comparing the intake of selected foods, alcohol and tobacco among groups.

^c^*P*-values for generalized linear models adjusted for sex, age, body mass index, smoking, alcohol drinking, regular exercise, family history of colorectal cancer, marital status, education level and household income as appropriate.

^d^*P*-values from Student’s *t*-tests comparing between genotypes of the dominant model (AA versus GG+GA).

**Table 5:** Mean consumption of selected foods, alcohol and tobacco for the combined TAS2R38 and CA6 genotype in all subjects and colorectal cancer cases and controls (mean ± standard deviation)^a^

TAS2R38+CA6^b^	Energy (kcal/day)	Vegetables (g/day)			Fruits (g/day)		Fiber (g/day)	Fat-food (g/day)	Sweets (g/day)	Alcohol^c^ (g/day)	Tobacco^c^ (cigarettes/day)
		All	Cruciferous	Dark green	All	Citrus					
All subjects (*n* = 2036)											
PAV/*+AA (*n* = 288)	2018.3 ± 658.0	367.3 ± 190.3	175.8 ± 106.9	36.7 ± 38.3	181.3 ± 197.2	43.8 ± 63.4	19.0 ± 6.4	4.8 ± 5.3	23.3 ± 28.6	24.3 ± 30.3	17.1 ± 10.1
PAV/*+G* (*n* = 1,419)	1967.2 ± 634.1	373.7 ± 188.4	178.4 ± 108.9	38.4 ± 34.4	184.3 ± 182.5	38.5 ± 53.1	19.5 ± 6.5	4.7 ± 4.6	26.1 ± 41.2	22.8 ± 31.1	16.6 ± 9.1
AVI/AVI+AA (*n* = 65)	2091.6 ± 678.8	406.5 ± 231.7	174.9 ± 118.8	41.1 ± 29.2	230.9 ± 305.8	44.2 ± 60.1	20.3 ± 6.8	3.8 ± 3.7	25.7 ± 36.5	18.0 ± 23.1	20.0 ± 13.4
AVI/AVI+G* (*n* = 264)	1947.1 ± 600.4	378.2 ± 188.0	178.6 ± 117.1	38.9 ± 29.3	187.6 ± 201.4	39.9 ± 52.7	19.8 ± 6.6	4.7 ± 4.7	28.1 ± 43.7	18.7 ± 24.0	15.7 ± 8.4
*p*_crude_^d^	0.293	0.612	0.907	0.112	0.188	0.776	0.316	0.324	0.877	0.240	0.125
*p*_adjusted_^e^	0.131	0.879	0.829	0.529	0.393	0.829	0.778	0.401	0.833	0.358	0.306
Controls (*n* = 1,356)											
PAV/* + AA (*n* = 169)	1811.1 ± 557.1	396.9 ± 215	188 ± 123.7	41.0 ± 43.2	197.1 ± 227.4	46.2 ± 73.2	20.2 ± 7.0	4.6 ± 5.4	21.3 ± 24.2	17.3 ± 22.8	15.2 ± 7.2
PAV/* + G/* (*n* = 950)	1841.2 ± 603.7	389.8 ± 205.7	183.5 ± 117.2	42.1 ± 38.4	197.7 ± 195.3	39.8 ± 55.9	20.3 ± 7.0	4.5 ± 4.6	25.5 ± 42.1	17.4 ± 21.9	15.8 ± 8.2
AVI/AVI + AA (*n* = 42)	1900.8 ± 645.0	412.9 ± 252.8	167.2 ± 108.9	43.8 ± 32.7	265.3 ± 362.9	45.7 ± 66.2	20.6 ± 7.4	4.4 ± 4.1	28.0 ± 40.4	13.0 ± 11.5	15.0 ± 5.1
AVI/AVI + G/* (*n* = 195)	1859.7 ± 593.3	393.2 ± 202.7	183.5 ± 127.4	41.6 ± 31.9	205.4 ± 218.4	42.6 ± 56.6	20.6 ± 7.0	4.4 ± 4.3	27.1 ± 44.3	17.5 ± 23.1	14.8 ± 8.8
*p*_crude_	0.846	0.942	0.854	0.649	0.231	0.525	0.951	0.998	0.791	0.890	0.217
*p*_adjusted_	0.726	0.959	0.319	0.952	0.540	0.319	0.929	0.998	0.777	0.750	0.246
Cases (*n* = 680)											
PAV/* + AA (*n* = 119)	2312.5 ± 680.1	325.3 ± 138.8	158.5 ± 74.1	30.7 ± 28.9	159.0 ± 141.7	40.2 ± 45.3	17.4 ± 5.0	5.1 ± 5.1	26.1 ± 33.8	34.0 ± 36.3	20.0 ± 12.9
PAV/* + G/* (*n* = 469)	2222.5 ± 617.9	341.3 ± 142.0	167.9 ± 88.9	31.0 ± 23.0	157.3 ± 150.3	35.9 ± 46.6	17.8 ± 5.0	5.2 ± 4.4	27.4 ± 39.2	34.2 ± 42.7	18.3 ± 10.3
AVI/AVI + AA (*n* = 23)	2439.9 ± 607.2	394.8 ± 192.0	189.0 ± 136.3	36.1 ± 21.1	168.1 ± 142.6	41.1 ± 46.3	19.7 ± 5.9	2.7 ± 2.8	21.5 ± 28.3	30.1 ± 37.3	30.0 ± 18.9
AVI/AVI + G/* (*n* = 69)	2194.2 ± 553.3	335.6 ± 129.9	164.7 ± 80.4	31.1 ± 18.6	137.3 ± 131.4	31.7 ± 37.7	17.6 ± 4.5	5.3 ± 5.8	31 ± 42.2	22.4 ± 26.6	18.5 ± 6.2
*p*_crude_	0.253	0.322	0.774	0.310	0.271	0.339	0.251	0.044	0.499	0.265	0.065
*p*_adjusted_	0.070	0.294	0.452	0.185	0.225	0.452	0.271	0.057	0.561	0.245	0.024

^a^Subjects with the AAV/AVI (*n* = 3), PAV/AVV (*n* = 1), PVI/AVI (*n* = 1), PVV/AVI (*n* = 1) diplotype were excluded from the statistical analyses due to the limited numbers.

^b^Combined genotype for the recessive and dominant models of the *TAS2R38* and *CA6* genetic variants, PAV/*=PAV/PAV+PAV/AVI, G* = GG + GA.

^c^The mean consumption was estimated among current/former drinkers and smokers.

^d^*P*-values for crude generalized linear models comparing the intake of selected foods, alcohol and tobacco among groups.

^e^*P*-values for generalized linear models adjusted for sex, age, body mass index, smoking, alcohol drinking, regular exercise, family history of colorectal cancer, marital status, education level and household income as appropriate.

**Figure 1:**
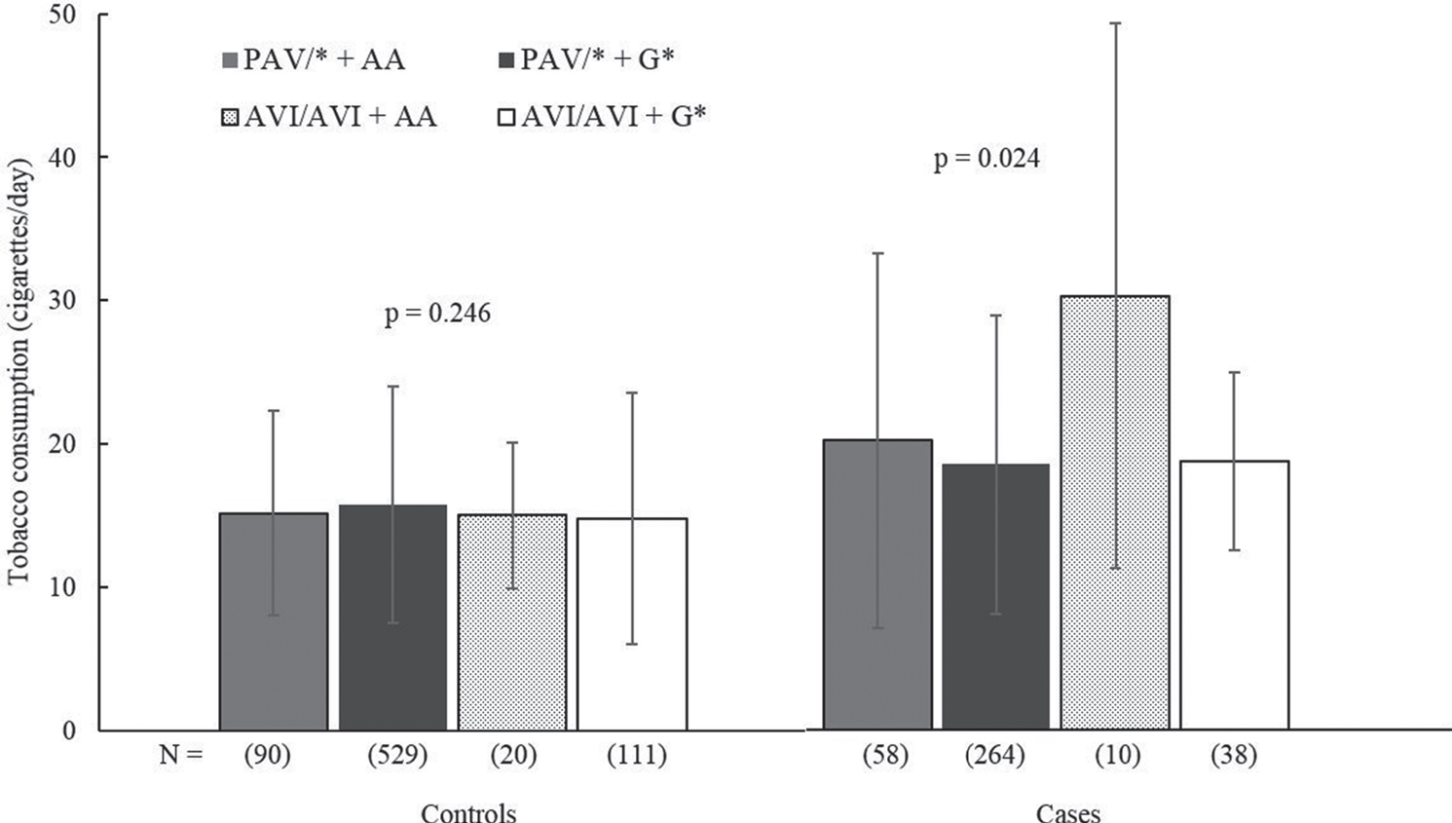
The consumption of tobacco cigarettes for the *TAS2R38-CA6* genotypes by colorectal cancer phenotype. Each bar presents mean ± standard deviation The mean consumption was estimated among current/former smokers. Numbers in brackets represent the numbers of subjects. *P*-values are from generalized linear models adjusted for sex, age, body mass index, alcohol drinking, regular exercise, family history of colorectal cancer, marital status, education level, household income and dietary zinc intake. No significant difference between pair of genotypes was estimated by Tukey's tests.

When the combined genotype of *TAS2R38* and *CA6* was analyzed, the results again showed that the genetic variants had no meaningful influence on the intake of the examined foods or alcohol in either all subjects or the controls (Table 5). However, among the CRC cases, the combined genotypes were associated with differential tobacco intake when confounding factors were considered (*p* = 0.024). Former/current smoking cases with the AVI/AVI ± AA genotype tended to be associated with higher daily tobacco use than the other genotypes, although the differences between genotypes were not clearly predicted by Tukey's test (Figure 1).

### The association between bitter taste genetic variants and the risk for colorectal cancer

The associations between the *TAS2R38* diplotype, the *CA6* rs2274333 genotype and CRC susceptibility are presented in Table 6. Our logistic regression models provided clear evidence that genetic variations in bitterness perception modify the risk for CRC independently, without any modifications in dietary intake. Having the *TAS2R38* AVI/AVI diplotype decreased the risk of CRC by approximately 25% compared to having the PAV haplotype [adjusted odds ratio (OR) = 0.74, 95% confidence interval (CI): 0.56–0.99]. *CA6* rs2274333 was also predicted to modify the risk of CRC: subjects with the G allele had a lower risk of developing CRC than those with the AA genotype (adjusted OR = 0.71, 95% CI: 0.55–0.92).

**Table 6:** The associations for the TAS2R38 diplotype, CA6 rs2274333 genotype and their combined genotype with risk for colorectal cancer

	OR_crude_^a^(95%CI)^b^	OR_adjusted_^c^(95%CI)	*p*	*p*_trend_^d^
*TAS2R38* diplotype				
PAV/PAV	1.00 (Reference)	1.00 (Reference)		0.064
PAV/AVI	0.99 (0.81–1.21)	0.94 (0.76–1.18)	0.600	
AVI/AVI	**0.74 (0.55**–**0.98)**	**0.70 (0.51**–**0.95)**	**0.022**	
PAV/* vs. AVI/AVI^e,f^	**0.74 (0.57**–**0.96)**	**0.74 (0.56**–**0.99)**	**0.021**	-
*CA6* rs2274333				
AA	1.00 (Reference)	1.00 (Reference)		**0.028**
GA	0.68 (0.52–0.87)	0.69 (0.52–0.90)	0.007	
GG	**0.73 (0.56**–**0.94)**	**0.73 (0.54**–**0.97)**	**0.034**	
AA vs. G*^g^	**0.70 (0.55**–**0.88)**	**0.71 (0.55**–**0.92)**	**0.008**	-
Combined genotype (*TAS2R38*+*CA6*)^h^				
PAV/*+AA	1.00 (Reference)	1.00 (Reference)		**0.006**
PAV/*+G*	**0.70 (0.54**–**0.91)**	**0.71 (0.53**–**0.94)**	**0.016**	
AVI/AVI+AA	0.78 (0.44–1.36)	0.73 (0.40–1.34)	0.312	
AVI/AVI+G*	**0.50 (0.35**–**0.72)**	**0.49 (0.34**–**0.74)**	**<.001**	

^a^Crude odds ratios (OR) from the logistic regression models for genetic variants and risk for colorectal cancer.

^b^95% confidence interval (CI).

^c^OR adjusted for sex, age, body mass index, smoking, alcohol drinking, regular exercise, family history of colorectal cancer, marital status, education level, household income and dietary zinc intake where appropriate.

^d^*P*-values for trend.

^e^vs.=versus.

^f^PAV/*=PAV/PAV+PAV/AVI.

^g^G*=GG+GA.

^h^Combined genotype for the recessive and dominant models of the *TAS2R38* and *CA6* genetic variants.

When *TAS2R38* and *CA6* variations were both taken into account, the combined genotype retained the protective effect of each variant allele/diplotype against CRC susceptibility (Table 6). Subjects with the *TAS2R38* PAV/* diplotype and the *CA6* variant G allele were less likely to have CRC (adjusted OR = 0.71, 95% CI: 0.53–0.94), compared to those with the reference PAV/* and AA genotype. This protective effect appeared to be greater when subjects possessed both the variant diplotype and the variant allele (adjusted OR for AVI/AVI+G* = 0.49, 95% CI: 0.34-0.74). Subjects with the AVI/AVI diplotype and the AA genotype also showed a reduced risk of CRC, though this result was not statistically significant.

## DISCUSSION

Genetic variations in taste perception mechanisms have been known to modify human dietary behavior and health outcomes. The current study examined whether genetic variants related to bitterness sensing are associated with the intake of dietary and consumer goods and the susceptibility to CRC in Koreans. Our findings suggested that the bitterness-related genetic variants did not influence the intake of the examined foods. However, these genetic variants seemed to be associated with the risk of CRC via other potential carcinogenic mechanisms and not by modifying dietary consumption.

Experimental studies have shown that genetic variants in *TAS2R38* and *CA6* are responsible for bitter taste sensitivity, *TAS2R38* variants have mainly been investigated to determine their modifying effects on dietary intake. To the best of our knowledge, this is the first study to use an epidemiological approach to examine whether CAVI is associated with the consumption of dietary foods and consumer goods. However, the findings suggested that the *CA6* rs22743333 genotype did not lead to any differential intake of dietary and consumer goods. Furthermore, the combination of the *CA6* rs22743333 genotype and *TAS2R38* variation also showed an ambiguous effect on the dietary and alcohol intake of Koreans. A similarly negligible influence of bitterness genetic variants on food intake was observed in our earlier study on gastric cancer and *TAS2R38*; in that study, the genetic variations had no significant influence on the population's dietary intake [11]. Though cruciferous vegetables are a major vegetable source for Koreans (48% of the total vegetable intake), these foods are generally consumed as pickled/salted dishes or as kimchi with multiple types of condiments, including salt, ginger, pepper, garlic, chili and fish sauce, which all have strong sapidity. The use of natural and artificial condiments (e.g., monosodium glutamate) may mask the native bitter flavor of cruciferous vegetables, and consequently, genetic variation in bitterness sensing might not influence the intake of bitter-tasting food. Additionally, the distribution of the combined *TAS2R38-CA6* variation may be associated with the minimal difference in the dietary intake between genotypes. *TAS2R38* and *CA6* reside on different chromosomes and their variations are independent (*P* = 0.221, chi-squared test, Supplementary Table 1). However, a majority of Koreans (69.7%) possess both the taster *TAS2R38* PAV and the non-taster *CA6* G allele. Having both variants may neutralize the contrasting effect of each allele on bitterness sensitivity, thus increasing the tolerance to bitter-tasting foods. Therefore, no clear differences in dietary intake may be observed among genotypes. Nonetheless, we should not underestimate the effects of bitterness-related genetic variants on dietary and consumer goods intake: in our data set, cases with *TAS2R38* AVI/AVI and the combined AVI/AVI-AA genotype tended to consume more tobacco than other genotypes. It is difficult to determine whether the presence of the variant allele was the main effector of the high tobacco consumption, as the association between *TAS2R38* AVI/AVI and tobacco intake was marginal. Furthermore, only small numbers of former/current smoking cases had the combined genotype (*n* = 10), and the effects of other confounding factors beyond genetic components may exist (the genotype-tobacco intake association was only predicted by the statistical model after adjusting for confounders). Despite these limitations, this evidence suggests that genetic modulation of bitterness intensity could influence an individual's intake of dietary and consumer goods by interacting with socio-economic characteristics and health behavior and, as a result, may potentially contribute to the risk of CRC [28]. Additional studies are required to establish a clearer role for taste-related genetic variations in the dietary and consumer goods intake of Koreans.

Though the effects of genetic variants on dietary intake were minimal, the *TAS2R38* and *CA6* genetic variations were associated with CRC outcomes on their own. This result supports the earlier findings that bitterness sensing-related variations in *TAS2R38* and *CA6* could be genetic markers of gastrointestinal function and disease [10, 15, 25]. When the effect of each genetic variation on the risk of CRC was evaluated, the *TAS2R38* AVI/AVI diplotype decreased the risk of CRC approximately 30% compared to the PAV haplotype. Because the AVI haplotype was associated with reduced bitterness intensity and an increased risk of CRC in a Czech-German population, the variant AVI/AVI diplotype has been suggested to be a potential biomarker for impaired gastrointestinal function [10]. However, another experimental study proved that the AVI/AVI diplotype is not simply a functional marker for the impaired T2R38 variant protein, as expression of the homozygous AVI transcript was detected, and individuals with the AVI/AVI diplotype were shown to respond to other bitter compounds [29]. Accordingly, it could be hypothesized that although the AVI variant barely responds to thiourea ligands, the structural perturbation of the AVI variant protein may enhance the sensing of other unknown carcinogenic molecules. Therefore, the AVI variant protein may have advantages in terms of signal transmission involved in the neutralization and expulsion of those unknown carcinogens from the intestine, thus reducing the risk of CRC. A similar protective effect of the AVI/AVI diplotype was also evident in another study: in Japanese-Americans, the AVI/AVI diplotype tended to be protective against CRC, although the power of the statistical model was limited [15]. The differential genotype distribution and the potential carcinogens in different dietary cultures and surrounding environments may have led to such a contrasting association between the *TAS2R38* genotype and CRC among different ethnic groups.

The pathogenic role of CAVI has been intensively investigated in relation to dental health because the CAVI protein is mainly secreted by the salivary glands. However, humans swallow large quantities of CAVI every day, and the physiological roles and associations of CAV1 with upper alimentary tract diseases have been previously observed [20, 25]. The current study also reveals that CAVI may be linked to various gastrointestinal diseases, including colorectal malignancies. *In silico* analyses predicted that the rs22274333 variant G allele leads to a critical structural modification of CAVI that limits the binding of zinc such that the GG variant protein may have reduced efficacy in catalyzing CO_2_ + H_2_O ↔ HCO_3_^–^ + H^+^ [26]. Because this variant protein may lead to increasingly acidic conditions in the digestive organs, individuals with the GG genotype are thought to be vulnerable to dental and ulcerative diseases. However, this rationale runs counter to the current findings, as individuals with the G allele had a decreased risk of CRC. Several hypotheses may be proposed to explain the contradictory outcomes among the studies. Although excessive acidity is considered a risk factor for alimentary disorders, gastric acidity is a decisive protective barrier from food- or water-borne toxic molecules [30]. Gastric acidity is also responsible for the composition of the microbiome in the vertebrate intestinal system, and the optimal gastric acidity may vary depending on dietary habits [30]. The potentially higher acidity of body fluids, including saliva and gastric juice, may benefit the alimentary tract by conferring protection against potentially carcinogenic compounds that Koreans are exposed to, thereby potentially reducing the risk of CRC. Additionally, the acidity of the gastric environment is critical for the stability/salvaging of folate vitamers. In acidic gastric juice (pH 3.5), 5-methyltetrahydfrofolate (the bioactive form of folate) is more stable than at a higher pH [31]. Moreover, the improved bioavailability of 5-methyltetrahydfrofolate may be associated with the sufficient provision of folate in the synthesis of pyrimidine and thymidylate, which could be involved in anti-carcinogenic mechanisms. Finally, CAVI is likely linked to the immune response, which may be associated with carcinogenic etiology. In a murine CAVI-deficient model, silenced CAVI expression resulted in the perturbation of lymphoid follicles in the intestinal Peyer's patches and the up- and down-regulation of 127 genes, which were mostly involved in catabolic processes in the duodenum [18]. Variant CAVI may lead to the perturbation of the immune system and related carcinogenic mechanisms and may, therefore, be linked to the risk of CRC. Cancer development and progression are multifactorial processes, and the role of CAVI in these processes has barely been explored. It was therefore difficult to elucidate the precise mechanistic relationship between the variant CAVI protein and the risk of CRC in this study. However, the current findings and the mechanisms speculated above could imply that variant CAVI plays a pathological role in CRC etiology. This hypothesis should be tested in future investigations.

Since T2R38 and CAVI show a functional overlap in bitterness perception, the role of each protein and their combined effects in bitterness sensing have been of particular interest in many studies. Evidence has suggested that the *TAS2R38* PAV haplotype is associated with the perception of higher concentrations of PROP, whereas the *CA6* A allele is relevant to sensing lower PROP concentrations [16]. However, a subsequent study reported that the variant CAVI was only associated with fungiform papilla density, whereas T2R38 modified bitterness sensing [32]. In addition to those observations regarding bitterness sensing, the present study adds more evidence for the combined effect of both T2R38 and CAVI in disease etiology. The combined genotype conferred the same protective effect of a single variant allele, and having both the variant allele and the variant haplotype resulted in an additive reduction in the risk of CRC. Unlike the PROP taste intensity study, the current observational study did not allow us to predict how either T2R38 or CAVI independently or cooperatively modify the risk of CRC. The modifying effect of the combined *TAS2R38*-*CA6* genotype on the risk of CRC may arise independently because these two proteins are mainly involved in different physiological metabolisms (signal transduction and maintaining homeostasis). However, both proteins are commonly responsive to thiourea moieties and are involved in energy metabolism and body homeostasis [33]. Additionally, T2R38 and CAVI are factors in the regulation of innate immunity, which is critical for the development and progression of CRC [34]. Considering our findings that the protective effect of the combined genotype against CRC increased with the numbers of variant alleles and haplotypes, the presence of mutually Supplementary roles or a potential mechanistic linkage between T2R38 and CAVI in CRC etiology (as well as bitterness sensing) cannot be discounted. More investigations with larger population sizes and different experimental approaches aimed at verifying the underpinning mechanism between T2R38 and CAVI are required.

This study provides new epidemiological evidence for the role of T2R38 and CAVI in the dietary intake of and CRC onset in Koreans. However, the findings must be interpreted with caution because some potential limitations may exist. First, this research employed a case-control study design; therefore, it may be affected by selection or recall bias in subject recruitment and data collection. Second, the validated self-reported FFQ applied in the current study may harbor potential systematic and random measurement issues in dietary evaluation. Third, the controls were enrolled on a volunteer basis from among participants of a health screening examination. These controls may be more concerned with a healthier lifestyle that is associated with a reduced risk of CRC, though we tried to minimize such differences by addressing confounding factors in the statistical models. Lastly, we examined the intake of various types of foods and consumer goods as well as the genetic variants known to crucially modify the activity of T2R38 and CAVI. However, other unexamined foods and polymorphisms linked to, or retained in, those genes may contribute to the dietary intake and CRC outcomes.

In conclusion, *TAS2R38* and *CA6* genetic variants in bitterness perception did not influence the dietary intake of Koreans. However, *TAS2R38* and *CA6* genetic variants were modifying factors of CRC susceptibility. This may indicate that the bitterness sensing receptors T2R38 and CAVI are involved in colorectal carcinogenesis and that their genetic variations are potential biomarkers for gastrointestinal function.

## MATERIALS AND METHODS

### Subject recruitment and data collection

**Figure 2:**
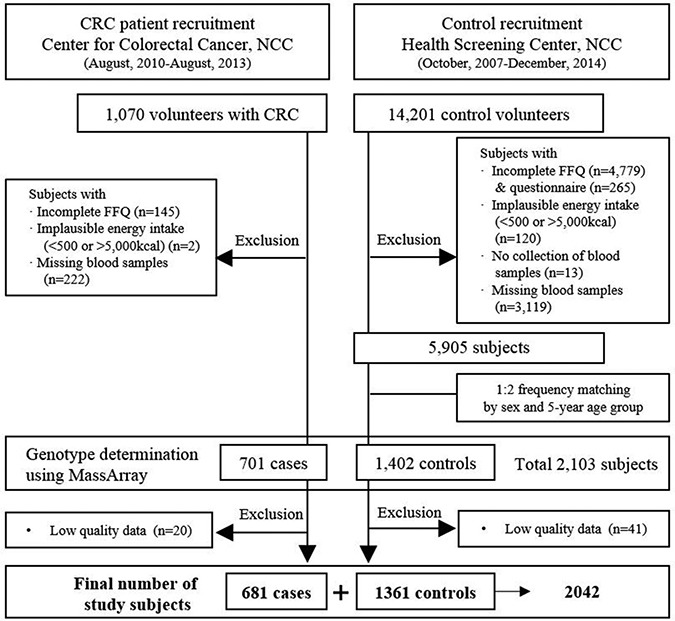
Simplified flow chart for the selection of study subjects CRC = colorectal cancer; NCC = National Cancer Center; FFQ = food frequency questionnaire.

This study was a part of CRC research conducted in National Cancer Center (NCC), Korea, from October 2007 to December 2014. The details of subject recruitment and data collection procedures were described previously [35]. Briefly, cases were defined as patients who underwent surgery for CRC or were histologically diagnosed with CRC at the Center for Colorectal Cancer, NCC. Controls were enrolled among visitors for a health screening examination (a benefit program of the National Health Insurance Cooperation) at the Center for Cancer Prevention and Detection, NCC. A total of 1,070 colorectal cancer patients and 14,201 controls volunteered for the study. However, individuals with incomplete descriptive and food frequency questionnaire (FFQ) data, unknown energy intake, or those with blood samples that were missing or could not be collected were excluded from the study (see Figure 2 for the details of the subject selection procedure). Among the remaining subjects, 701 cases and 1,402 controls were selected for the study at a 1:2 frequency and matched by sex and 5-year age group, and their genotypes were determined. Finally, 681 cases and 1,361 controls with qualified genotypic data were analyzed for the study. Prior to the commencement of the study, all study procedures were approved by the ethical committee of NCC (NCCNCS-10-350 and NCC2015-0202) and the actual study was carried out following approved protocols.

### Dietary intake analyses

Participants were requested to complete the validated FFQ [36]. The FFQ presented three portion sizes and nine degrees of frequency for each food item, and participants were asked to describe their food intake over the last 12 months. Dietary intake was analyzed using CAN-PRO 4.0 (Computer Aided Nutritional Analysis Program, The Korean Nutrition Society, Seoul, Korea). The major focus of the present study (the effect of genetic variants on dietary intake) was evaluated from the following ten groups of food and nutrients: total energy, all vegetables, cruciferous vegetables, dark green vegetables, all fruits, citrus fruits, fiber, fat-food, sweets and zinc. Additionally, subjects’ daily consumption of alcohol (g/day) and tobacco products (cigarettes/day) were also determined using a structured questionnaire (see Supplementary Table 2 for the investigated food items and alcoholic beverages in each category).

### Genotyping and genetic data analyses

Genotypes of *TAS2R38* (A49P, V262A and I296V) and *CA6* (rs2274333) were assessed using the Agenabio MassArray iPLEX^®^ gold assay (San Diego, CA, USA). The primary data were analyzed using Agenabio TYPER version 4.0, and the raw results were only accepted as a qualified genotype if the call rate for each locus was over 95%. The fundamental genetic analyses were conducted using Haploview (Version 4.2). The diplotypes of *TAS2R38* were computed using FAMHAP software [37].

### Statistical analyses

The general characteristics of the study population were compared based on CRC phenotype using Student's *t*-tests and chi-squared tests. Differences in the distribution of *TAS2R38* diplotypes and *CA6* genotypes were evaluated using chi-squared tests. All dietary data were analyzed after adjusting for the total energy intake using Willet's residual method [38] and log-transformation. Analyses of variance were employed to evaluate the difference in food and consumer goods intake between genotypes and diplotypes in the presence or absence of potential confounding factors. Tukey's tests were employed for post hoc comparisons. The logistic regression models were established to predict the association between risk of CRC and genetic variations and denoted as OR with a 95% CI. All statistical analyses were performed using SAS version 9.3 (SAS Institute Inc., Cary, NC, USA). A two-sided *P-value* of less than 0.05 was considered statistically significant.

## SUPPLEMENTARY MATERIALS TABLES


