# Supplementary material for: Variations in the bitterness perception-related genes TAS2R38 and CA6 modify the risk for colorectal cancer in Koreans
Source: Oncotarget. 2017 Feb 19;8(13):21253–65. doi: 10.18632/oncotarget.15512 (PMC5400581; doi:10.18632/oncotarget.15512)
Supplement: Supplementary file 1 [file oncotarget-08-21253-s001.pdf]

Variations in the bitterness perception-related genes *TAS2R38* and *CA6* modify the risk for colorectal cancer in Koreans

Supplementary Materials

Supplementary Table 1: Distribution of the *TAS2R38* diplotype by *CA6* rs2274333 genotype (%)

		CA6 rs2274333			p^a
		AA	GA	GG	
TAS2R38 diplotype	PAV/PAV	126 (17.4)	335 (46.1)	265 (36.5)	0.221
	PAV/AVI	162 (16.5)	467 (47.6)	352 (35.9)	
	AVI/AVI	65 (19.7)	152 (46.2)	112 (34.1)	
	AAV/AVI	-	2 (66.7)	1 (33.3)	
	PAV/AVV	-	-	1 (100.0)	
	PVI/AVI	-	-	1 (100.0)	
	PVV/AVI	-	1 (100.0)	-	

[a]*P*-value from the chi-square test examining the association between the *TAS2R38* and *CA6* variants.

Supplementary Table 2: The groups of foods and alcoholic beverages analyzed in the current study

Group	Food and alcoholic beverage items
All vegetables	all vegetables
Cruciferous	white radish, radish leaves, mustard, mustard leaves, napa cabbage, broccoli, cabbage and other cruciferous
Dark green	curled mallow, chicory, pumpkin leaf, pine leaf, sweet potato vines, hot pepper, hot pepper leaf, perilla leaf, field dropwort, angelica, water parsley, chives, lettuce, iceberg lettuce, celery, spinach, mugwort, crown daisy, taro vine and others
All fruits	all fruits
Citrus fruits	mandarin, cumquat, orange and orange juice
Fatty foods	margarine, butter, beef fat, sesame oil, coffee whitener and soybean oil
Alcoholic beverages	beer, hard liquor, Korean spirits, Korean rice wine, wine and fruit liquor
